# Legal Notes for Institutional Workers

**Published:** 1912-03-09

**Authors:** 


### LEGAL NOTES FOR INSTITUTIONAL WORKERS.

**(The Editor will be pleased to answer Legal queries in this column.)**

### The Interfering Committee-man.

In order to avoid congestion, and to facilitate the despatch of business in hospitals and other institutions, it is customary for the work of the general committee to be divided amongst a number of sub-committees. A division of labour is thus effected, but sometimes a member of the General Committee desires to interfere in the proceedings of a sub-committee to which he does not belong. Strictly speaking, and unless there is a special rule to prevent it, any committeeman may attend the meetings of any sub-committee. This is so, at any rate, in the case of a local authority. If an over-zealous person insists on enforcing his legal rights, what is the chairman to do? Adjournment of the proceedings involves delay; violent ejection might lead to an action for assault, or a " swearing of the peace." An effective and wholly peaceable method was adopted not long since by the chairman of a committee of a local authority not far from London. A Socialist member of the Council attended in assertion of his right to be present. The chairman and other members took their seats at the table. In accordance with a preconcerted scheme, no one spoke. No business was transacted. After about fifteen minutes the Socialist saw that he was merely delaying the proceedings, and left. Nor could he afterwards assert that he had been refused admittance or that he had been turned out!

### Hospitals and the Companies Act.

To facilitate management, and to enable the Liverpool Dental Hospital more conveniently to conduct its affairs and hold property in its own right instead of having to resort to the cumbrous method of a trust deed, the committee have decided that it would be desirable to incorporate the hospital under the Companies Act, 1908, with the licence of the Board of Trade to dispense with the word " Limited " in its title. The proposal having been approved at a general meeting in November last, the necessary steps are now being taken to obtain registration. In what circumstances does the Board of Trade allow the word " Limited " to be omitted from the name of a limited liability company? The word is placed there to warn the public that the liability of shareholders is limited to the amount of their shares; and it is obvious that if the company is not a trading concern in the ordinary sense of the term, the risk to which persons dealing with it are exposed is very small.

Yet if the word is omitted the Board of Trade prohibits the payment of dividends to members, and orders the company to provide by its memorandum that in the case of liquidation the surplus assets shall not be distributed amongst the members, but shall be given to some other institution having similar objects. Another limitation is that such a company without a Board of Trade licence cannot hold more than two acres of land.

### Use of Houses as Sanatoria.

The owner of a private dwelling is sometimes confronted with the problem—Can I use my house as a sanatorium or nursing home? If he owns the freehold, it is difficult to see how this form of user can be prevented; but in the case of leasehold premises, a restrictive covenant may intervene to prevent it. In a case heard in 1880 the lease of a dwelling-house contained a covenant that the lessee should not do anything which should be a "nuisance or annoyance " to the neighbourhood. The lessee proposed to convert the dwelling-house into a sanatorium. It was held that to use the house as a sanatorium would be to make it, if not a nuisance, an annoyance. In another case a lease which prohibited the carrying on " of any trade, business, or dealing whatsoever, or anything in the nature thereof," was assigned to the committee of management of a hospital for diseases of the throat and chest. Patients made small payments according to their means. It was held that the covenant had been broken by the use of the house as a hospital.

### The Right to Operate on an Infant.

It is strange that the legal questions which arise in relation to operations on infants have never yet been discussed in any English Court. In private practice, of course, no difficulty arises because the parent or guardian can be consulted beforehand. The consent of a person *in loco parentis* would clearly justify the surgeon who undertakes the most serious operation. But suppose the parent cannot be found or is not available in an urgent hospital case, can the operator proceed without incurring liability to an action for damages? The only case which throws any light on the subject was heard last year at Toronto. A youth of nineteen, who was suffering from goître, attended at the Toronto hospital. He was advised to undergo an operation, and as he appeared willing to do so he was moved to a surgical ward, where one half of the thyroid was removed. He made a good recovery and went home; but his parents subsequently brought suit against the surgeon for having performed the operation without their consent or that of the patient. Mr. Justice Falconbridge, in giving judgment, said that there appeared to be no case which established the proposition that in a case such as this the parents' consent must be obtained. He then continued: " I shall not be the first to declare there is such a law. Of course if it was the case of an infant of tender years, or the case of a person manifestly of weak intellect, it might be another matter; but this young man, while manifestly not of the brightest intelligence, is apparently well enough able to take care of himself." In the result, the defendant was entirely vindicated. There is little doubt that our English Courts would follow this decision. Persons under twenty-one are infants in the eye of the law; but it is a rule which is sometimes honoured in the breach.

